# Cardiac imaging procedures and the COVID-19 pandemic: recommendations of the European Society of Cardiovascular Radiology (ESCR)

**DOI:** 10.1007/s10554-020-01892-8

**Published:** 2020-05-26

**Authors:** Dietrich Beitzke, Rodrigo Salgado, Marco Francone, Karl-Friedrich Kreitner, Luigi Natale, Jens Bremerich, Matthias Gutberlet, Ellie Mousseaux, Konstantin Nikolaou, Charles Peebles, Birgitta Velthuis, Rozemarijn Vliegenthart, Christian Loewe, Tilman Emrich, Executive Committee of the European Society of Cardiovascular Radiology (ESCR):, Bremerich Jens, Natale Luigi, Gutberlet Matthias, Vliegenthart Rozemarijn, Nikolaou Konstantin, Francone Marco, Loewe Christian, Velthuis Brigitta, Salgado Rodrigo, Peebles Charles, Mousseaux Ellie

**Affiliations:** 1 Department of Biomedical Imaging and Image-Guided Therapy, Medical University of Vienna, Wien, Austria; 2 Department of Radiology, Antwerp University Hospital & Holy Heart Hospital, Antwerp/Lier, Belgium; 3 Department of Radiological, Oncological and Pathological Sciences, Sapienza University, Policlinico Umberto I, Rome, Italy; 4 Department of Diagnostic and Interventional Radiology, University Medical Center of the Johannes Gutenberg University Mainz, Mainz, Germany; 5 Department of Radiological and Haematological Sciences - Institute of Radiology, Catholic University of Rome, Fondazione Policlinico Universitario Gemelli - IRCCS, Rome, Italy; 6 Department of Radiology, University Hospital Basel, Basel, Switzerland; 7 Department of Radiology, Heart Center Leipzig, University of Leipzig, Leipzig, Germany; 8 Department of Radiology, Assistance Publique-Hôpitaux de Paris, Hôpital Européen Georges Pompidou, Paris, France; 9 Department for Diagnostic and Interventional Radiology, University Hospital Tuebingen, Tuebingen, Germany; 10 Department of Cardiothoracic Radiology, University Hospital Southampton, Southampton, UK; 11 Department of Radiology, University Medical Center Utrecht, Utrecht, The Netherlands; 12 Department of Radiology, University Medical Center Groningen, Groningen, The Netherlands; 13 Division of Cardiovascular Imaging, Department of Radiology and Radiological Science, Medical University of South Carolina, Charleston, SC USA; 14 German Centre for Cardiovascular Research (DZHK), Partner Site Rhine-Main, Mainz, Germany

**Keywords:** SARS-Cov-2, COVID-19, Cardiac magnetic resonance, Cardiovascular computed tomography, Safety

## Abstract

The severe acute respiratory syndrome coronavirus 2019 (SARS-CoV-2) pandemic currently constitutes a significant burden on worldwide health care systems, with important implications on many levels, including radiology departments. Given the established fundamental role of cardiovascular imaging in modern healthcare, and the specific value of cardiopulmonary radiology in COVID-19 patients, departmental organisation and imaging programs need to be restructured during the pandemic in order to provide access to modern cardiovascular services to both infected and non-infected patients while ensuring safety for healthcare professionals. The uninterrupted availability of cardiovascular radiology services remains, particularly during the current pandemic outbreak, crucial for the initial evaluation and further follow-up of patients with suspected or known cardiovascular diseases in order to avoid unnecessary complications. Suspected or established COVID-19 patients may also have concomitant cardiovascular symptoms and require further imaging investigations. This statement by the European Society of Cardiovascular Radiology (ESCR) provides information on measures for safety of healthcare professionals and recommendations for cardiovascular imaging during the pandemic in both non-infected and COVID-19 patients.

## Introduction

The severe acute respiratory syndrome coronavirus 2 (SARS-CoV-2) leading to the current Coronavirus disease (COVID-19) pandemic is putting high pressure on healthcare systems in several countries in both hemispheres. The start and progression of the pandemic varies between regions and countries, with some countries outside China currently making phased and tightly-regulated attempts to ease previously imposed social restrictions. However, many experts argue that this disease may be part of public life for many months to come, requiring a long-term plan to cope with a varying influx of patients and protection of healthcare workers while awaiting the development of effective pharmacological treatment [1, 2]. Therefore, radiology departments need to remain vigilant to cope with this pandemic for the near future.

As known, COVID-19 is a highly infectious disease submitted through small droplets with a prodromal phase (varying in intensity and length) preceding the onset of potential severe symptoms in the majority of patients. As such, the resulting large cohort of non- or mildly symptomatic infected people accelerates spreading of the disease and complicates triage between infected and non-infected patients. While COVID-19 is predominately characterised by a wide range of respiratory symptoms, neurological, gastrointestinal and cardiovascular symptoms have been described as well at various stages of the disease [3–5].

Given the established and fundamental diagnostic role of cardiovascular imaging in modern healthcare, and the specific value of cardiopulmonary radiology in COVID-19 patients, departmental organisation and imaging programs need to be restructured during the pandemic in order to provide access to modern cardiovascular services while ensuring safety for healthcare professionals and other patients. The uninterrupted availability of cardiovascular radiology services remains crucial particularly during the current pandemic outbreak, to establish a correct diagnosis and to avoid unnecessary complications in different patient populations.

While suspected or established COVID-19 patients may have concomitant cardiovascular symptoms and require further imaging investigations, non-infected patients with pre-existing or acute cardiac events still must be granted access to cardiac imaging in order not to underdiagnose or delay treatment of relevant cardiovascular disease. Therefore, potential non-infected patients with cardiovascular symptoms should still be encouraged to present to the hospital during the pandemic despite the current social restrictions, and a pathway for clinical evaluation and imaging examinations should be provided.

Finally, current imaging protocols should be tailored to the specific situation and provide a fast and comprehensive approach to detect cardiothoracic involvement in COVID-19.

This paper represents a knowledge and experienced-based expert opinion on how to ensure continuous availability of cardiac imaging during the current COVID-19 pandemic. It may have to be tailored to the local resources, workflow and hygiene guidelines and the characteristics of national and regional healthcare systems during the outbreak.

## Restructuring the radiology department

Protecting patients and healthcare professionals at all levels must be the main goal while providing high-quality imaging services during this pandemic. Reports from Italy during the outbreak indicate that around 10% of the subjects who tested positive for Corona Virus were healthcare professionals, with a death toll of around 150 medical doctors by the end of April 2020 [6].

Therefore, (re)structuring of the departmental workflow to ensure optimal and save pathways to imaging modalities is of tremendous importance to both patients and healthcare professionals.

### Access to scanners, equipment disinfection and technical considerations

Radiology departments and their different imaging modality rooms are not designed to be used during a viral pandemic outbreak. Nevertheless, they deliver an important contribution to COVID-19 disease diagnosis and management, and must subsequently adapt to keep functioning under the current unusual conditions and intense pressure [7].

#### Scanner access

If possible, suspected or confirmed COVID-19 patients should be imaged in dedicated COVID-19 X-ray, CT and MR equipment in the radiology department in order to prevent cross-contamination between infected and non-infected patient populations. Some hospitals have even resorted to the expedited new installation of such dedicated CT scanners for the exclusive purpose of investigating (potential) COVID-19 patients [8]. If this is not possible e.g. due to a limited number of available primary scanners, other scanner types (e.g. SPECT-CT scanners or the cone-beam function of angiography suites) could be used as “COVID-19” patient scanners for general purpose use. The objective would be to either divert (potentially) infected patients to a specific scanner as previously mentioned, or to increase capacity on other CT-scanners who are better suited for cardiovascular applications, depending on the local situation. Notably, all scan protocols on non-routine CT-scanners (e.g. SPECT-CT scanners) must be adapted to provide sufficient diagnostic quality, for which close cooperation and coordination with industrial partners might be helpful.

Finally, if a dedicated CT-scanner for infected patients is not available, at least dedicated COVID-19 time slots on the available equipment must be defined, preferable at the end of the day. These timeslots can also be made available for the investigation of (potential) COVID-19 patients who require high-quality cardiovascular CT-imaging, which may not be available on the dedicated COVID-scanner.

Access routes to these scanners must be highlighted to transport services and clinicians, and also clearly indicated to non-radiology staff. These routes, as well as the corresponding waiting areas, must be separated from normal patient pathways.

#### Equipment disinfection

The SARS-CoV-2 virus has been reported to survive a variable amount of time on several types of surfaces and environmental conditions [9, 10]. Cleaning the imaging facility after every (suspected) positive patient before scanning a negative patient is mandatory and must be performed according to local guidelines. Conversely, cleaning between scanning of two (or even more) established SARS-CoV-2 positive patients is not necessary unless (bacterial) superinfection is known, and local guidelines may differ. Currently, there are no universally accepted guidelines on how to clean radiology imaging equipment for SARS-CoV-2, although some centres have published their experience, proposing different methods and solutions for disinfecting the several components of CT and MR examination rooms [8]. Lists of disinfectants effective against SARS-CoV-2 have been published (https://www.epa.gov/pesticide-registration/list-n-disinfectants-use-against-sars-cov-2), while a solution of 62–71% ethanol, 0.5% hydrogen peroxide or 0.1% sodium hypochlorite within 1 min of possible contamination has also been suggested [11]. Additionally, several manufacturers have published equipment-specific guidelines for disinfection of their imaging equipment (Table 1). Down-time for disinfection between patients varies according to the local available resources and also on the targeted imaging equipment. The immediate availability of “cleaning” teams for scanners with mixed populations of SARS-CoV-2 positive and negative patients is advised to achieve maximum safety and productivity.

**Table 1:** Vendor's recommendations for disinfection of scanners and scanner facilities (in alphabetical order)

Vendor	Website
Canon	https://eu.medical.canon/covid-19/
General Electric	https://cleaning.gehealthcare.com/
Phillips	https://www.usa.philips.com/healthcare/medical-specialties/covid-19/precision-diagnostics-addressing-covid#education_and_resources
Siemens	https://www.siemens-healthineers.com/en-be/clinical-specialities/critical-care/disinfection-recommendations https://www.siemens-healthineers.com/en-ph/services/proper-disinfection

If possible, the scanner facilities should be ventilated under low pressure as compared to surrounding rooms (similar concept as for septic surgical areas) to ensure that potential contaminated aerosols are cleared via the climate controls and do not contaminate scanner control rooms or the corridor.

### Protecting imaging professionals

Healthcare professionals represent the prototype of a possible “super spreader” due to the multiple contacts with colleagues and patients. Consequently, protection of all healthcare professionals in a radiological department (radiographers, nurses, radiologists and other medical staff) from potential infection and subsequent transmission to patients and colleagues must be ensured in order to maintain a continuous medical service to the patients in need. This involves multiple organisational entities beyond the imaging department, especially hygiene, allied disciplines and transport services.

Furthermore, a standardized hospital-wide communication protocol must be implemented when requesting imaging services for established and potentially infected patients in order to avoid misunderstandings and maintain separated in-department pathways for both patient populations, preventing staff and patient nosocomial infections. Some departments have e.g. established a dedicated phone number at the radiology department to request such examinations, with a special digital-only pathway for the transfer of forms from COVID-19 wards.

#### Resources

Personal Protective equipment (PPE) for maintaining a safe patient service onsite is of the highest importance, however the global market for these products is currently under pressure. Therefore, local health care authorities and professional bodies should be encouraged to help and assist in the buying process of PPE. PPE must include a high number of various types of masks ranging from simple surgical masks for general staff to highly protective FFP2 masks for technicians and doctors with direct contact with COVID-19 patients. Protective gowns, eyeglasses and eye shields also need to be provided. In Table 2 we provide an overview on which kind of PPE should be used during imaging procedures. To avoid wastage of these precious products all levels of healthcare professionals involved in imaging procedures must be repeatedly taught in the correct use of them [12]. Finally, many hospitals make the wearing of protective masks mandatory to all hospital personnel and incoming visitors.

**Table 2:** Categories and use of PPE for cardiac imaging procedures in COVID-19 positive patients

Use of PPE	Health care professionals		Patient
COVID-19 confirmed or suspected (unkown)	Patient contact	No direct patient contact	
CCTA/CMR	Full set of PPE including FFP2 mask, gloves, gown, goggles and/or face shield	FFP2, (gown)	Mouth nose protection
Emergency with the need of intubation	Evacuate room with the exception of the emergency team	Do not enter scanner room	N/A
COVID-19 negative inhouse	Patient contact	No direct patient contact	
CCTA/CMR	FFP2 mask, gloves, glown	FFP2 or Mouth nose protection	Mouth nose protection

#### Hygiene

Imaging staff must be trained in the correct use of PPE and other measures of infection control. Mistakes during dressing and especially undressing are the main source for contamination of the staff and the surrounding area. Furthermore, instructions in basic hygiene like handwashing should be implemented. Local hygiene protocols must be strictly implemented into the clinical workflow with appropriate training [12].

#### Working in teams/cohorts

During the pandemic, outpatient throughput should be decreased with cancellation of many elective investigations in order to avoid cross-contamination between in- and outpatients and to refocus inhouse resources. If possible, work schedules of imaging professionals should be reorganized in teams that are working completely separated. Teams of radiographers and radiologists should be changed simultaneously if possible. This measure is of high importance to decrease the potential impact of quarantine measures on the department if a cohort member of the staff might be infected. (Non-essential) meetings must be cancelled and/or shifted to virtual online meetings. If absolutely necessary, meetings should be reduced to a minimum of participants and should be held ensuring adequate distance of more than 1.5 m in a sufficiently large meeting room. When such reconfigurations of the radiology department have been applied in association with protection and education as well as training of personnel, some reports indicate that radiology staff can be effectively protected against SARS-CoV-2 infections [13].

#### Testing of healthcare professionals

Reverse transcription polymerase chain reaction (RT-PCR) testing of a nasopharyngeal mucosal swab is currently the reference standard for proving an active Sars-CoV-2 infection, also used in mild or even asymptomatic patients. While RT-PCR has a very high specificity making a positive test pragmatically diagnostic, its reported sensitivity varies between 60 and 70%, leading to a potential 30–40% of false-negative results (also due to other factors). Given their multiple patient contacts under sometimes difficult conditions, healthcare imaging professionals are especially at risk for infection and might also transfer this infection to vulnerable patients or colleagues. To avoid this pathway of spreading, and its potential catastrophic impact on patients and hospital departments, high vigilance for potential infections of staff members is necessary.

RT-PCR testing policies vary between hospitals, regions and countries depending on several factors. In many hospitals, the RT-PCR test is not indicated for asymptomatic staff members, and consequently routine swab testing of asymptomatic hospital personnel for screening purposes is not performed. Given the indicated lower sensitivity of RT-PCR test, a negative result does not necessarily equate to being free of infection. Possible strategies for follow-up of healthcare professionals who have been in contact with infected patients (in- or outside the hospital) include self-screening for fever twice a day and vigilance for respiratory symptoms. Once a healthcare worker shows any clinical sign of infection, he must be removed from the staff pool for further investigation.

Antibody tests—also known as serological tests—that detect the immune response to an existing infection are not yet reliable enough and widely available but are currently being developed and prepared for mass deployment. They have the potential to play a role in this complex situation. Results from these tests can help identify who has been infected and has developed antibodies that may protect them from future infections as well as identify those still at risk. Nevertheless, as many questions regarding the individual immune response to Sars-CoV-2 infection remain unresolved, current knowledge is too weak to recommend specific guidelines regarding the timing, use and interpretation of antibody tests.

#### Telemedicine

Social distancing and home working solutions have proven to be efficient barriers against viral spread. Therefore teleradiology, if possible, should be implemented to ensure safety of the imaging staff. Remote reporting within the hospital is also beneficial for working in smaller cohorts, with people at home as back-up to in-house staff. Care must be taken in terms of data protection and technical setup of the outsourced teams.

## Cardiac Imaging procedures

### Cardiovascular implications of COVID-19 disease and the rationale for non-invasive CT-MR imaging

Several investigators have reported on the involvement of the cardiovascular system in COVID-19 patients. However, at this particular moment in time there is a lack of large multicentre studies and fundamental research to establish the exact mechanism of disease and effect of COVID-19 disease on the cardiovascular system. Nevertheless, several reports point to an exacerbated inflammatory response with myocardial injury and heart failure, and an increased risk in the presence of pre-existing disease.

The presence of pre-existing cardiovascular disease in COVID-19 patients significantly increases their reported death rate (10.5%) compared to other pre-existing conditions like diabetes or cancer [14]. This is important not only as COVID-19 patients with pre-existing cardiac disease form a large proportion of the symptomatic patient population, but also as exacerbation of pre-existing cardiovascular diseases and secondary cardiac involvement in case of systemic spreading of the infection may further compromise prognosis [15–17].

In a recent single-centre analysis, almost 20% of hospitalised COVID-19 patients had cardiac injury, i.e. elevation of troponin I levels, associated with an increased complication rate and need for mechanical ventilation compared to COVID-19 patients without cardiac involvement [18]. Myocardial injury has recently been significantly associated with fatal outcomes in COVID-19 patients in several series [17, 19]. In patients with COVID-19 pneumonia, an age ≥ 65 years, pre-existing cardiovascular disease or cerebrovascular disease but especially the presence of CD3 + CD8 + T cells ≤ 75 cell µL^−1^ and elevated cardiac troponin I ≥ 0.05 ng mL^−1^ have been identified as important predictors of mortality [20].

This and other publications report increased troponin levels in association with ECG abnormalities indicating cardiac injury in COVID-19 patients. A meta-analysis based on 4 studies with a total of 341 patients demonstrated a significant difference in cardiac troponin levels between subjects with severe and non-severe COVID-19 disease [21]. A main cause of high troponin levels and myocardial injury could be represented by acute myocarditis of varying severity. In a few cases the occurrence of myocarditis in COVID-19 has been reported but rarely associated with cardiac dysfunction. A recent autopsy study has revealed significant viral RNA in the heart (among other organs) exceeding levels of viremia [22]. This data suggests that SARS-CoV-2 spreads of the bloodstream and can infect other organs. Additionally, myocarditis has been associated in the past with other coronavirus diseases as MERS [23, 24]. In a case series of 150 patients, 7% of deaths were reported as related to acute heart failure consequent to myocarditis [16]. Nevertheless, the exact relation between the presence of the SARS-CoV-2 virus and the pathogenesis of myocardial damage is not fully understood and further investigation is necessary. Increased troponin levels could also be related to acute type II myocardial infarction (mismatch between oxygen supply and demand without unstable coronary artery disease) or to plaque rupture caused by severe inflammation (type I myocardial infarction). Such a relation between inflammation and cardiovascular events have been previously reported in influenza and in other coronavirus infections [25].

Heart failure has been reported in 23% of COVID-19 patients in one study [26], but case fatality rate among those with pre-existing comorbidity was related to cardiovascular disease in 10.5% in China[15]. To date, it remains unclear if fatality is caused by acute myocardial injury or exacerbation of a pre-existing cardiac disease. Cardiogenic pulmonary oedema is relatively common in this situation, but the differential diagnosis with ARDS is not always easy, even using imaging and Berlin criteria [27]. Nevertheless, a cardiogenic component of the acute failure is important when establishing an ECMO procedure.

Abnormal coagulation parameters have been reported; particularly, increased d-Dimer is associated with a worse prognosis [26]. Disseminated intravascular coagulation, vascular inflammation and prolonged immobilization may contribute to an overall increased thromboembolic risk. Particularly in patients with rapid clinical worsening and hypoxia, pulmonary embolism has to be considered and CT pulmonary angiography may be performed for further investigation [28].

### Indications for cardiac imaging in suspected or confirmed COVID-19 patients

Non-invasive CT or MR cardiac imaging indications during the pandemic should be balanced between continuously maintaining the best possible care and the ability to perform these procedures safely, correctly and at the most appropriate time. As such, good interdisciplinary communication is essential as in-house conditions and possibilities invariably differ between different centres.

Categorising the assignments into vital emergency situations, urgent indications and elective indications should be considered using an interdisciplinary approach. Elective patients with non-urgent indications can be postponed to a later time point if clinically possible. Furthermore, the choice of the modality is relevant for healthcare professionals and patient safety. One must consider that echocardiography (and especially transoesophageal echocardiography, which is a high-risk procedure like intubation) cannot be performed with keeping a distance of at least one meter to the patients and/or is requested as a bedside test on the ICU. CT and MR on the other hand also need patient preparation and cannulation which require patient contact with radiographers and nurses [29]. Transportation and contamination of MR and CT scanners also should be considered as a source of potential infection. Finally, the length of the examination also must be optimised as this is also associated with potential virus contamination of the scanner systems and well as reduced patient tolerance in those who are sicker and find supine imaging difficult to maintain.

#### Cardiac Computed tomography angiography (CCTA)

Atrial fibrillation and other arrhythmic events may occur in the course of the disease due to fever, hemodynamic stress or volume (over)load to prevent renal failure. Ruling out left atrial appendage thrombus by transesophageal echocardiography prior to cardioversion in this setting appears to be of high risk for the performing physician (because of the need for intubation of the probe in the patient). Additionally, the procedure is stressful for the patient who may also suffer from shortness of breath due to the underlying disease. Therefore, CCTA including a late phase may be considered as a non-invasive procedure in these patients [30]. The same holds true for evaluation of valve endocarditis where CCTA should also be considered as an alternative to transoesophageal echocardiography, but should be carefully read in close correlation with clinical circumstances [31].

As previously explained, patients with COVID-19 can develop raised troponin levels especially in an advanced stage of disease with an yet unclear mechanism of myocardial damage or minor thromboembolic events associated to a hypercoagulable state [32]. Therefore, in case of pre-existing or unknown but suspected cardiac disease, exclusion of coronary artery disease (CAD) with CCTA is helpful to avoid cardiac catheterization, the latter being a procedure which under the current conditions is more challenging than usual. This crisis might be the moment to expand the utilization of CCTA to detect CAD in clinical reality, as suggested by the current ESC guidelines who consider CCTA a class I level A indication in patients with chronic coronary syndromes irrespective of age [33].

Additionally, coronary CTA should be considered in COVID-19 patients presenting with acute chest pain and raised troponins without ST-elevation at ECG to rule out CAD, addressing a possible myocarditis/pericarditis or Tako-Tsubo disease, and as such avoid unnecessary invasive coronary angiography which can be at risk for the operator.

Furthermore, the ruling out of pulmonary embolism should be provided in COVID-19 patients because their increased risk of venous thromboembolism and the common presentation of elevated D-Dimers, hypoxia and dyspnea [26]. If clinical and cardiac symptoms remain unclear, triple rule-out protocols—even if they often do not provide perfect diagnostic statements for all diagnostic relevant targets—for the evaluation of the pulmonary circulation and the coronary arteries might also be considered [16].

In the assessment of aortic stenosis in the need for surgical or non-surgical aortic valve replacement (TAVR), CCTA might be used as an alternative to pre-interventional angiography in an emergency or urgent situation and should be used for device selection and assessment of valve composition [34–36].

CCTA in general also provides insights into lung structure and -pathologies in COVID-19 patients which is especially relevant in patients with moderate to severe respiratory symptoms or to screen for complications, but does not include all lung fields [19]. Therefore, and depending on local equipment, it may be considered to add an additional low-dose scan of the whole lung before or after CT imaging of the heart [37]. Non-gated low-dose CT (preferably with i.v. contrast medium) can also be helpful to detect cardiac abnormalities, e.g. right ventricular- or left ventricular dilatation or intracardiac thrombi. Furthermore, as with every heavily diseased patient, COVID-19 patients could be more affected by side-effects of medication usually administered for cardiac-CT (e.g. beta-blockers). We want to underline the possibility of an interdisciplinary discussion with the referring physician to prevent such adverse events due to side-effects.

#### Cardiac magnetic resonance (CMR)

Reports are increasing that CMR can detect COVID-19 associated myocarditis. As a hypercoagulable state is also reported associated with this disease, a potential differential diagnosis to myocarditis could be Myocardial Infarction with Non-obstructive Coronary Arteries (MINOCA). Furthermore, literature has reported cases of stress-induced cardiomyopathy (Tako-Tsubo syndrome) in co-existence with COVID-19 [38]. Independent of the SARS-CoV-2 pandemic, differentiating MINOCA from myocarditis and other causes of acute myocardial injury is possible using CMR and therefore impacts patient management for secondary prevention [20]. Additionally, CMR has the potential to incidentally detect extracardiac findings (e.g. lung involvement, pleural effusion) which may be clinically relevant.

However, direct diagnostic and prognostic implications of CMR imaging in the specific SARS CoV−2 pandemic are actually not known. Therefore, the usefulness of CMR might be limited for the diagnosis and differentiation between myocarditis, Tako-Tsubo syndrome and MINOCA. Additionally, CMR is more time-consuming than CT and therefore poses a potential higher risk for patients and staff (e.g. because of viral transmission). To overcome this obstacle, this statement would propose a fast and focused CMR protocol for a comprehensive assessment of cardiac involvement in COVID-19. This protocol incorporates CINE sequences for functional assessment, oedema imaging for detection of acute myocardial injury and T1 mapping as well as late gadolinium enhancement (LGE) imaging for differentiation between ischemic and non-ischemic causes of myocardial injury. This protocol should be accompanied by fast, one breath-hold sequences for assessment of thoracic complications. All efforts to reduce acquisition time (e.g. use of accelerated imaging as compressed sensing or reduction of time delay for LGE imaging) should be used. A proposed imaging protocol is summarized in Table 3.

**Table 3:** Fast and accelerated CMR protocol for comprehensive CMR assessment in suspected cardiac involvement of COVID-19

Assessment	Sequence	Comment
Function	CINE	Use acceleration (e.g. compressed sensing), limited number of acquisitions, acquisition after contrast injection/before LGE
Oedema	T1/T2 Mapping/T2 STIR	Mapping preferred to T2-STIR because of image quality, use limited representative SA slices for screening
Scar	3D-PSIR (SA)	LGE imaging after 6 min post contrast administration; adjust contrast media protocol
Thoracic	T2	One breath-hold sequences for lung pathology

SA : Short Axis

Indications for cardiac imaging are summarized in Table 4.

**Table 4:** Possible indications for cardiac imaging during the COVID-19 pandemic

CCTA	CMR
COVID-19 positive patients	
Detection of LA or LAA Thrombus in atrial fibrillation	Acute Myocarditis, DD MINOCA, Tako-Tsubo Syndrome)
Rule out of coronary artery disease	
Rule out of pulmonary embolism (ev. Triple rule out protocols)	
Detection of suspected valvular endocarditis	
TAVI planning	
COVID-19 negative patients in-house	
Pre-operative CTA	Acute Myocarditis, DD MINOCA, Tako-Tsubo Syndrome)
Rule out of coronary artery disease	Dilated Cardiomyopathy
	(Stress imaging)

### Indications of cardiac imaging of non-suspicious or confirmed COVID-19 negative patients

#### Outpatients

COVID-19 related impact on hospital and departmental organisation varies between regions, countries and types of hospitals, with some hospitals in heavily affected regions accommodating a large proportion of COVID-19 patients making them potentially less able to handle outpatients. Nevertheless, the aim should always be to continuously provide safe cardiovascular CT- and MR imaging services in close collaboration with the referring clinician.

During the pandemic, all cardiac imaging procedures that are planned in an elective setting should be evaluated carefully, their planning adjusted to in-house healthcare guidelines, imaging capacity and clinical urgency. This is valid for CCTA as well as CMR. Indications for cardiac imaging during the pandemic must have an immediate impact on patient management as the imaging procedure interrupts social distancing and potentially put the SARS-CoV-2 negative patient at risk for infection.

### Inpatients

CT and MR imaging procedures of confirmed COVID-19 negative inpatients may be performed during the inhouse period in order not to delay diagnosis and prevent the patient from undertreatment of a medical condition. However, depending on the pandemic-imposed burden on the local hospital and its evolution over time, proper interdisciplinary communication is necessary to ensure that these examinations are performed at the most adequate moment for patient and staff in order to ensure overall safety and availability of properly disinfected equipment. Some hospitals may have more ability to continue delivering CT/MR imaging services than others.

Therefore, indications for CCTA include all kind of preoperative CCTA scans including TAVR, coronary artery bypass, (adult) congenital heart disease, cardiac transplant and rule-out of coronary heart disease as well as pulmonary embolism in the chest pain triage. Indications for CMR should be tailored to local capacities and the possibility for safe imaging of SARS-CoV-2 negative patients. In case for both conditions are fulfilled, patients should undergo normal standard of care and indications may include the workup of acute myocarditis, MINOCA and decompensated heart failure with reduced ejection fraction of unknown aetiology. Stress testing may be considered as an alternative to cardiac catheterization.

## Performing cardiac imaging programs after the first wave of the pandemic

Restoring the cardiac imaging program to pre-pandemic levels of activity is (and will be) challenging as long as there is no established vaccination for SARS-CoV-2. Safety of healthcare professionals and patients must be warranted, but at the same time a higher patient turnover will be mandatory as many of examinations were initially rescheduled. In the first step, patients with “subacute” indications for imaging in CCTA and CMR that were rescheduled should be scanned. This should be done in close accordance to the referring physicians (e.g. pre-operative examinations). Pre-operative scans may be scheduled close to the operations where an up-to-date COVID-19 testing will be available. If the number of these patients seems to be manageable, the program might be reopened to routine outpatients. Preferably, these patients should be contacted in advance in order to have more recent information on their wellbeing. Patients with suspected COVID-19 symptoms should either be tested prior to imaging or rescheduled again.

The use of PPE in this phase may be strongly recommended both for patients and for radiographers, nurses and radiologists, but guidelines may vary depending on the local situation. Patients should be wearing a surgical mask whereas radiographers and nurses in contact with patients should maintain all necessary precautions. In CMR, PPE has to be free of ferromagnetic or conducting materials in terms of MR Safety. Nevertheless, questions remain regarding the availability and distribution of PPE material to both hospital staff and the general population in many regions.

Cardiac Imaging and especially CMR might have an important role after the pandemic as myocardial involvement and changes in myocardial structure by scar induction and consecutive left ventricular dilatation might have an impact on the patient’s prognosis after surviving a COVID-19 episode. Additionally, it is currently not clear which impact COVID-19 will leave on lung structure as this might influence right ventricular function and size by the induction of pulmonary hypertension. Early evaluation of this patient cohort by CMR and CT might be not only of scientific interest it might also have an immediate impact on patient therapy.

##  Conclusion

Cardiac imaging during and after the initial phase of the COVID-19 pandemic is challenging. Patient and healthcare professionals safety must have the highest priority and therefore PPE resources must be provided. Training and teaching the use of PPE and hygiene procedure on adherence to the actual CDC and WHO guidelines, working in separated teams and if possible the use of telemedicine is of essential importance to avoid virus spreading in health care professionals.

Cardiac imaging services should help to avoid or at least cut down the number of risky invasive or time consuming procedures like cardiac catheterization or transoesophageal echocardiography. Therefore CCTA might be favoured in ruling out coronary heart disease and left atrial (appendage) thrombus and should be offered to COVID-19 positive patients when needed. CMR may deliver insights into myocardial involvement in SARS-CoV 2 patients and discriminate types of myocardial damage. Due to an associated hypercoagulable state ruling out pulmonary embolism by CT must also be offered to clinicians. Based on local capacities and possibilities to ensure safe imaging, non SARS-CoV-2 infected patients should undergo cardiac imaging when indicated to prevent undertreatment of cardiac conditions.

Setting up cardiac imaging program after the first, critical phase of the pandemic will have to be tailored to the need of allied disciplines and focused on the safety of patients and healthcare professionals.
